# Metabolomics study in severe extracranial carotid artery stenosis

**DOI:** 10.1186/s12883-019-1371-x

**Published:** 2019-06-24

**Authors:** Tsong-Hai Lee, Mei-Ling Cheng, Ming-Shi Shiao, Chia-Ni Lin

**Affiliations:** 1 Stroke Center and Department of Neurology, Linkou Chang Gung Memorial Hospital, Taoyuan, Taiwan; 2 Department of Medical Biotechnology and Laboratory Science, College of Medicine, Chang Gung University, Taoyuan, Taiwan; 3 Department of Biomedical Sciences, College of Medicine, Chang Gung University, Taoyuan, Taiwan; 4 Metabolomics Core Laboratory, Healthy Aging Research Center, Chang Gung University, Taoyuan, Taiwan; 5 Department of Laboratory Medicine, Linkou Chang Gung Memorial Hospital, Taoyuan, Taiwan

**Keywords:** Carotid stenosis, Metabolomics, Choline, Lipids, Atherosclerosis

## Abstract

**Background:**

Significant genetic association has been found in patients with severe carotid artery stenosis (CAS). The present study wished to investigate if metabolites may also act as biomarkers for CAS.

**Methods:**

Consecutive patients with at least one carotid artery stenosis > = 60% on cerebral angiography were prospectively recruited from May 2007 to January 2016. Normal controls were recruited from outpatient clinic who had no stroke and coronary artery disease (CAD) history, and the brain magnetic resonance or computed tomographic angiography showed bilateral CAS < 30%. Risk factor profile, clinical characteristics, age, and clinical features were recorded. All subjects were male, and none had diabetes. ^1^H-NMR spectroscopy-based metabolomics analysis was carried out for plasma samples.

**Results:**

Totally, 130 male subjects were recruited. Age had no significant difference between the controls and CAS group (60.2 ± 5.9 vs. 63.3 ± 6.0, *p* = 0.050). The CAS group had significantly higher frequency of CAD, hypertension, smoking and alcohol but lower body mass index than the controls (*p* < 0.05). The laboratory tests showed CAS group had significantly higher level of homocysteine but lower levels of cholesterol, high-density lipoprotein and hemoglobin than the controls (*p* < 0.05). The ^1^H-NMR based plasma metabolomics analysis indicated that choline was significantly lower in CAS patients. The VI*P* values of lipids were greater than 1.0, which were considered significantly different.

**Conclusions:**

Our results suggest homocysteine, choline and lipids in association with traditional risk factors may be involved in the pathogenesis of CAS. Diet adjustment to control homocysteine, choline and lipids may be helpful for the prevention of CAS.

**Electronic supplementary material:**

The online version of this article (10.1186/s12883-019-1371-x) contains supplementary material, which is available to authorized users.

## Background

Stroke is the third leading cause of death [1] in 2013 and the most common cause of disability worldwide. Due to the aging and growth of the population, there is an increase in global cerebrovascular deaths [2]. Cerebrovascular mortality is increasing if the combats against vascular risk factors are not successful [3]. This condition suggests there is a strong need to improve the prevention of vascular death.

Metabolomics has been applied in several fields of atherosclerosis research, including stable coronary artery disease (CAD) [4–6], acute coronary syndrome [7–9], atherosclerotic plaque composition [10, 11], diabetes [12], cerebral infarction/ischemia [13, 14], and cardiovascular surgery [15], etc. Nevertheless, there is no metabolomics report in patients with severe carotid artery stenosis (CAS). Previous genome-wide association studies [16, 17] including ours [18] have found HDAC9 (encoding histone deacetylase 9) on chromosome 7p21.1 and variants on chromosome 6p21.1 are associated with extracranial large artery atherosclerosis. It is possible that some specific metabolites can be related to the pathogenesis of extracranial CAS.

To improve our knowledge of the pathophysiological changes in atherosclerosis, the study of biomarkers related to atherogenesis is becoming increasingly important to reach an early detection of CAS. Metabolomics can be a good tool in the analysis of disease mechanisms and biomarkers of disease [19]. As metabolomics is defined as a useful study of small-molecule metabolites derived from cell metabolism, metabolomics can offer a snapshot of the cellular changes that are taking place before or during atherosclerosis progression using a biological sample. It is possible that metabolomics study can help to obtain the metabolite profiles that may play a certain role in the atherogenesis of CAS and identify those patients who will be at risk of or have suffered from atherosclerosis. The present study focused on the metabolomics study in association with traditional risk factors to investigate the biomarkers in patients with severe CAS.

## Methods

The study protocol was performed according to our previous report [20] and approved by the institutional review board of Chang Gung Memorial Hospital, Linkou Medical Center. The written informed consents were obtained from all subjects before their involvement in the study.

### Subjects

We prospectively recruited consecutive patients who were admitted for cerebral angiography (cerebral digital subtraction, brain computed tomographic or brain magnetic resonance angiography) to evaluate the necessity of carotid artery intervention and who had at least one of the carotid artery with diameter stenosis > = 60% in any segment from common carotid to extracranial internal carotid artery in our hospital during the study period from May, 2007 to January, 2016. The control group was recruited from the outpatient clinic, who had no stroke and CAD history, and the brain magnetic resonance or computed tomographic angiography showed bilateral carotid artery diameter stenosis < 30%. The diameter stenosis of artery was calculated by NASCET criteria [21]. Age, clinical presentations, risk factor profile, and laboratory blood tests were recorded (Table 1). Exclusion criteria included (i) the presence of systemic diseases such as hypothyroidism, decompensated liver cirrhosis, and systemic lupus erythematosus; (ii) the presence of other disorders such as cancer and severe infection that might compromise survival within 6 months; (iii) patients with modified Rankin scale > = 3; (iv) patients with a serum creatinine > = 2 mg/dL; and (v) patients with severe peripheral artery disease.

**Table 1:** Comparison of clinical profiles between controls and patients with severe extracranial carotid artery stenosis (CAS)

	Group		
	Control	CAS	*p* value
	*n* = 65	*n* = 65	
Age (years)	61.3 ± 5.2	63.2 ± 5.7	0.050
Male, n (%)	65 (100)	65 (100)	
Ischemic stroke, n (%)	0 (0)	63 (97.0)	< 0.001
Coronary artery disease, n (%)	4 (6.2)	19 (29.2)	< 0.001
Risk factor profile			
Hypertension, n (%)	25 (38.5)	48 (73.8)	< 0.001
Atrial fibrillation, n (%)	0 (0)	0 (0)	
Smoking, n (%)	25 (38.5)	52 (80.0)	< 0.001
Alcohol, n (%)	14 (22.5)	26 (40.0)	0.039
Hx of recurrent stroke, n (%)	0 (0)	18 (27.7)	< 0.001
Body mass index (kg/m^2^)	25.7 ± 7.0	24.6 ± 6.0	0.058
Laboratory blood test			
Homocysteine (mg/dL)	10.3 ± 4.0	12.9 ± 7.8	0.011
HS-CRP (mg/dL)	2.5 ± 5.8	3.2 ± 3.3	0.446
Total Cholesterol (mg/dL)	209.1 ± 43.2	178.9 ± 50.5	< 0.001
Triglyceride (mg/dL)	146.5 ± 78.0	135.5 ± 77.6	0.418
LDL-C (mg/dL)	123.2 ± 41.8	112.5 ± 38.8	0.105
HDL-C (mg/dL)	48.0 ± 13.2	38.9 ± 10.9	< 0.001
Serum sodium (mEq/L)	141.2 ± 47.0	140.3 ± 68.3	0.219
Hemoglobin (g/dL)	14.7 ± 7.4	13.7 ± 5.9	0.018
AC sugar (mg/dL)	100.7 ± 36.6	97.6 ± 49.4	0.304
Uric acid (mg/dL)	6.6 ± 1.9	6.3 ± 2.3	0.358
eGFR (ml/min/1.73 m^2^)	75.2 ± 28.1	84.0 ± 46.3	0.319

Patients with diabetes are excluded from the study. *LDL-C* low density lipoprotein-cholesterol, *HDL-C* high density lipoprotein-cholesterol, *eGFR* estimated glomerular filtration rate

### Blood sampling and examination

Blood samples were collected at enrollment for controls and in stationary phase (more than 3 months after stroke onset) for stroke patients. Samples for metabolomics were collected in sodium citrate tubes. Blood was centrifuged immediately (10 min, 3000 rpm at 4 °C) and plasma was aliquoted into separate polypropylene tubes that were immediately stored at -80 °C. Plasma was analyzed by metabolomics workflow described in the succeeding section. Measurement of other parameters, including homocysteine, C-reactive protein, lipid profiles, hemoglobin, blood sugar, and kidney function was conducted at Department of Laboratory Medicine in Chang Gung Memorial Hospital.

#### NMR analysis of the plasma

^1^H-NMR spectroscopy-based metabolomic analysis was carried out for plasma samples. The frozen plasma samples were thawed on the ice. An aliquot of 350 μL of plasma sample was mixed with 350 μL of a buffer solution (75 mM Na_2_HPO_4_, 0.08% TSP, 2 mM NaN_3_, 20% D_2_O). The mixture was centrifuged at 12,000×g at 277 K for 5 min. Finally, 600 μL of the supernatant was transferred to 5-mm NMR tubes for analysis. Quality control sample was prepared by pooled patient samples with one control sample in every 50 test samples [22].

^1^H-NMR spectra were acquired on a Bruker Avance II HD 600-MHz NMR spectrometer at 310 K using a 5-mm inverse triple resonance CryoProbe (^1^H/^13^C/^15^N) with cold preamplifier for ^1^H and ^13^C with a z-axis gradient (Bruker Biospin GmbH, Rheinstetten, Germany). The Carr-Purcell-Meiboom-Gill (CPMG) spin-echo pulse sequence (RD-90°-[τ-180°-τ] _n_-ACQ) was routinely applied. A total T_2_ relaxation time of 80 ms was used to attenuate broad signals from proteins and lipoproteins. The ^1^H NMR spectrum was collected with a spectral width of 12,019.23 Hz, relaxation delay of 4.0 s, and acquisition time of 2.7 s. Water solvent signal was suppressed by a continuous wave irradiation on water frequency during relaxation delay. Free induction decay (FID) was acquired into 72 k data points, and the FID acquisitions were accumulated 32 times to increase signal-to-noise ratio. FIDs were weighted by an exponential function with a 0.3 Hz line broadening factor prior to Fourier transformation. All acquired NMR spectra were phase- and baseline-corrected, then referenced to the doublet at 5.23 ppm.

All NMR spectra were phased and baseline-corrected using Topspin software (version 3.2.2; Bruker Biospin GmbH, Rheinstetten, Germany). Each ^1^H NMR spectrum from plasma was segmented into equal widths (0.01 ppm), corresponding to regions 9.50–0.50 ppm. The regions between 6.50 and 4.50 ppm containing residual water signal were removed. The spectral data were normalized to the reference.

### Statistical analyses

The resulting datasets were analyzed with statistical models including multivariate analyses principal-components-analysis (PCA), partial-least-squares-discriminant-analysis (PLS-DA), and orthogonal-projection-to-latent-structure-discriminant-analysis (OPLS-DA) using a web-based metabolomics tool, MetaboAnalyst (https://www.metaboanalyst.ca/MetaboAnalyst/faces/home.xhtml), and Umetrics SIMCA Version 13 (https://umetrics.com/products/simca) to find variables that were correlated across the samples. PCA, an unsupervised pattern recognition method, was performed to examine the intrinsic variation in the dataset. OPLS-DA was used to maximize covariance between the measured data (peak intensities from the NMR spectra) and the response variable (predictive classifications). A non-overlapping ^1^H signal from each metabolite was used to calculate the integral area, and the identified metabolites were quantified by relative peak intensity. The variable importance in the projection (VIP) value of each variable in the model was calculated to indicate its contribution to the classification. A higher VIP value represented a stronger contribution to discrimination among groups. The VIP values of those variables greater than 1.0 were considered significantly different.

The clinical results were expressed as the mean ± SD for continuous variables and as the number (percentage) for categorical variables. Data were compared by two-sample or paired t-tests and Chi-square, when appropriate. All statistical analyses were 2-sided and performed using SPSS software (version 15.0, SPSS, Chicago, IL, USA). The data with *p-values* < *0.05* were *considered significant*. SPSS software was also used to test the normality of control and CAS groups. The assessment for normality used Kolmogorov-Smirnov test. Of the 25 metabolites, 15 metabolites were normally distributed in control group and 13 in CAS group.

## Results

A total of 130 male subjects were recruited during the study period. Among these 130 patients, there were 65 control subjects and 65 CAS patients. As presented in Table 1, there was no significant difference in age between the controls and CAS group (60.2 ± 5.9 vs. 63.3 ± 6.0, *p* = 0.050). The CAS group has significantly higher frequency of CAD, hypertension, and smoking than the controls (*p* < 0.001). There was no stroke history in the control group but 97% of CAS patients had ischemic stroke and 28% had history of recurrent stroke. The laboratory blood tests showed CAS group had significantly lower levels of cholesterol and high-density lipoprotein than controls (*p* < 0.001). The homocysteine levels in CAS patients were higher than that in controls (*p* = 0.011), but the CRP levels did not have significant difference.

**Fig. 1:**
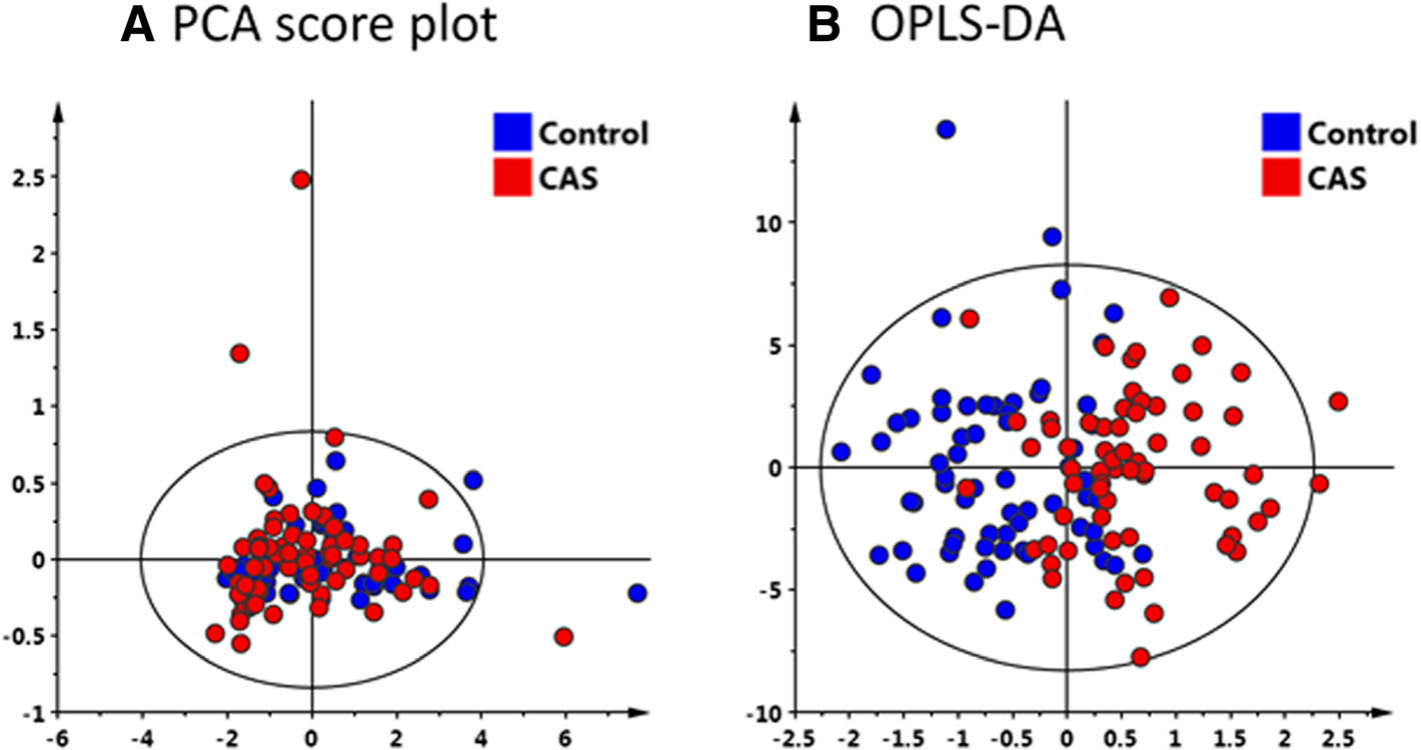
**a** Principal components analysis (PCA) is performed on the dataset containing the chemical shift of detected metabolites in plasma. On a PCA score scatter plots (**a**), each dot represents a sample (carotid artery stenosis [CAS] patient in red and control in green). The dot distribution shows homogeneous in each group, indicating no significant variation within group. The PCA score plot corresponding to the first two principal components (PC) shows that PC1 and 2 can explain 88.4 and 3.8%, respectively, of the dataset total variance. **b** To confirm the patterns observed in PCA and to identify metabolites responsible for these patterns, orthogonal-partial-least squares-discriminant-analysis (OPLS-DA) models are constructed to relate metabolic profiles to lipids, lactate, and choline. The 2-D score plot of metabolites in plasma samples between controls and CAS patients is shown in panel B which enables differentiation of controls and CAS patients

**Fig. 2:**
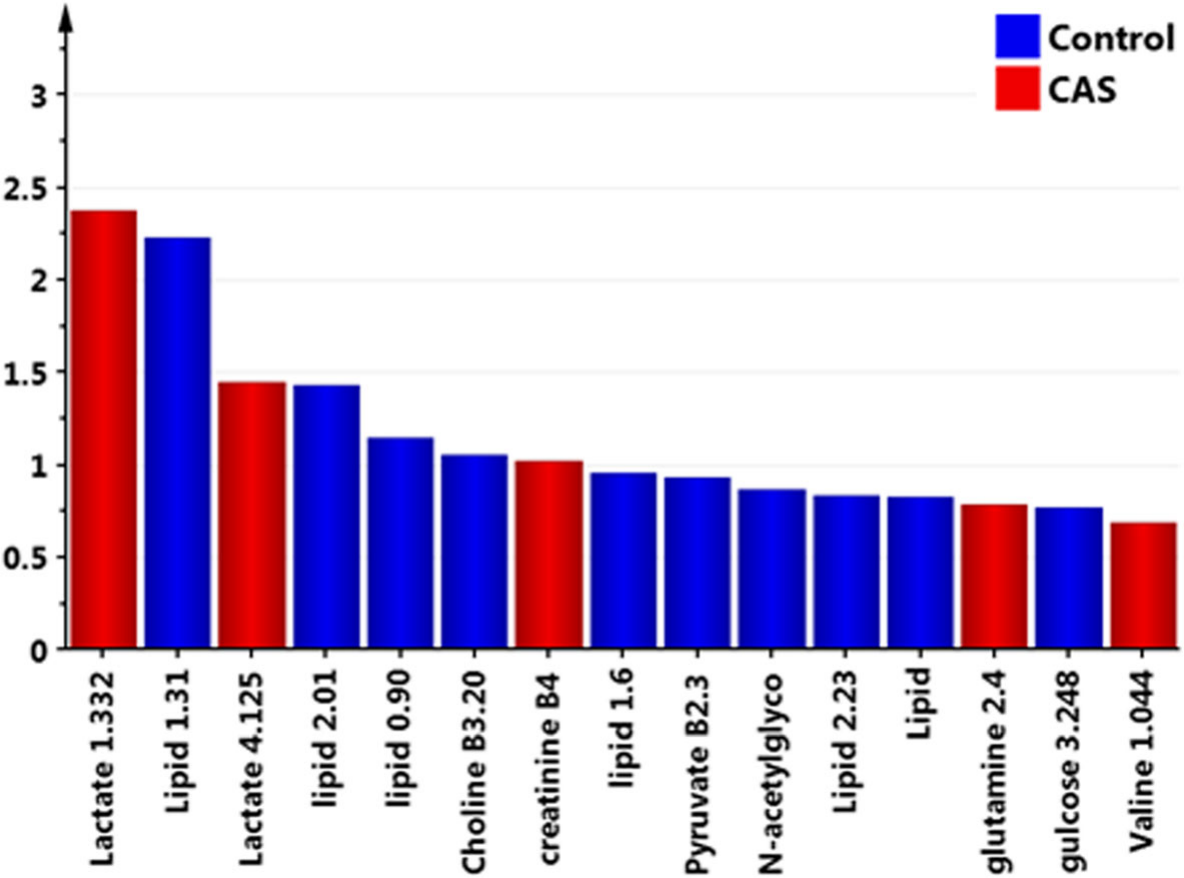
Analysis of the control and carotid artery stenosis (CAS) patients’ sample utilizing Umetrics SIMCA Version 13 reveals differences. The top 15 important features of the metabolomics markers identified by the scores of variable importance in the projection (VIP) are listed. Blue square represents controls, and red square represents CAS patients. The color of the bar indicates that the group of individuals has relatively high concentration comparing to the other group. When VIP scores of the metabolites are greater than 1.0, these metabolites are considered significantly different. In the PLS-DA model, we search 5 components for classification, and the VIP values in component 5 for lactate 1.332, lipid 1.31, lactate 4.125, lipid 2.01, lipid 0.90, choline B3.205, and creatinine B4.045 are 2.3771, 2.2358, 1.4470, 1.4364, 1.1465, 1.0570, and 1.0274, respectively

**Fig. 3:**
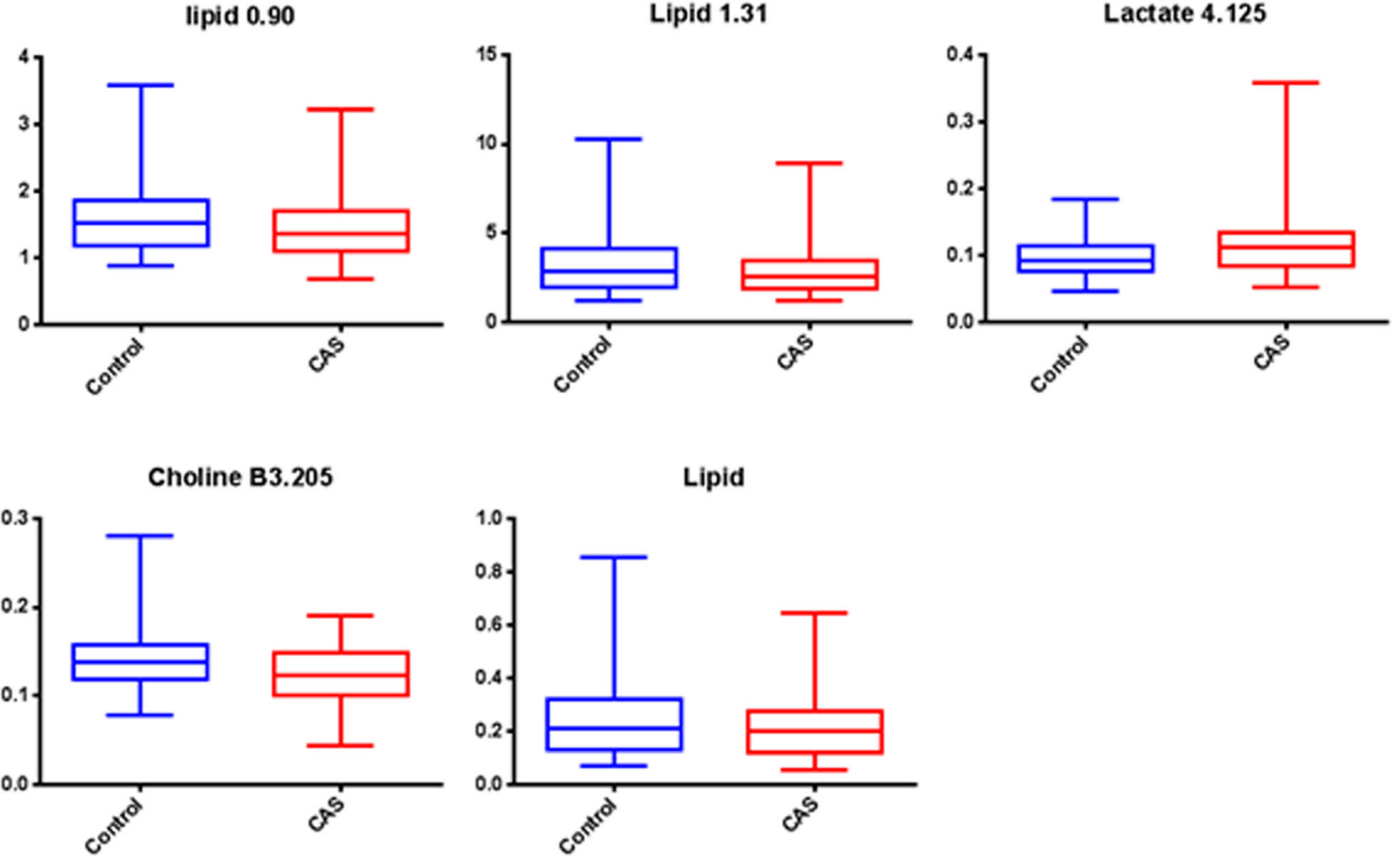
Box and Whisker plots show the distribution of the five potential metabolites to differentiate carotid artery stenosis (CAS) patients and controls. The ends of the box are the upper and lower quartiles, so the box spans the interquartile range. The median is marked by a line inside the box

The metabolomics approach with ^1^NMR as a tool was carried out on the plasma samples of CAS patients and control subjects. The ^1^H-NMR based plasma metabolomics analysis is an untargeted and semi-quantitated approach. The data were showed in Table 2, and the chemical shift of each metabolites were presented as mean ± SD. After data normalization and statistical analysis by t-test, the results indicated that choline was significantly lower in CAS patients (*p* = 0.00177). PCA was performed on the dataset containing the chemical shift of detected metabolites in plasma. On a PCA score plot (Fig. 1a), each dot represents a sample (CAS patient in red and control in green). The dot distribution was homogeneous in each group, indicating no significant variation within group. The PCA score plot (Fig. 1a) corresponding to the first two principal components showed that PC1 and 2 explained 88.4 and 3.8%, respectively, of the dataset total variance. In order to confirm the patterns observed in PCA and to identify metabolites responsible for these patterns, supervised OPLS-DA models were constructed to relate metabolic profiles to lipids, lactate, and choline. The 2-D score plot of metabolites in plasma samples between controls and CAS patients was shown in Fig. 1b. When VIP scores of the metabolites were greater than 1.0, these metabolites were also considered significantly different (Fig. 2). In the PLS-DA model, we searched 5 components for classification, and the VIP values in component 5 for lactate 1.332, lipid 1.31, lactate 4.125, lipid 2.01, lipid 0.90, choline B3.205, and creatinine B4.045 were 2.3771, 2.2358, 1.4470, 1.4364, 1.1465, 1.0570, and 1.0274, respectively. Box and Whisker plots of these potential metabolites to differentiate CAS patients and controls were shown in Fig. 3. The S-plot for OPLS-DA run by MetaboAnalyst 4.0 in control and CAS groups was also examined (Additional file 1: Figure S1).

**Table 2:** Comparison of the results of ^1^H NMR spectrum between controls and patients with severe extracranial carotid artery stenosis (CAS)

	Chemical shift (ppm)		
Metabolite ID	Control (*n* = 65)	CAS (*n* = 65)	*P* value
Formate (8.46)	0.00235 ± 0.00046	0.00222 ± 0.00049	0.12583
Histidine (7.778)	0.00756 ± 0.00185	0.00764 ± 0.00167	0.80616
Phenyalanine (7.38)	0.00767 ± 0.00134	0.00781 ± 0.00132	0.56561
Phenyalanine (7.336)	0.01426 ± 0.00204	0.01485 ± 0.00243	0.13568
Tyrosine (7.20)	0.01159 ± 0.00179	0.01168 ± 0.00218	0.79284
Histidine (7.056)	0.00743 ± 0.00166	0.00726 ± 0.00145	0.53744
Tyrosine (6.906)	0.01066 ± 0.00164	0.01020 ± 0.00197	0.15681
Lactate (4.125)	0.09552 ± 0.02848	0.11547 ± 0.04951	0.00584
Creatinine (4.045)	0.02056 ± 0.00705	0.03025 ± 0.03457	0.03006
Glucose (3.248)	0.18253 ± 0.03440	0.16856 ± 0.04925	0.06328
Choline (3.205)	0.14156 ± 0.03223	0.12382 ± 0.03109	0.00177
Glutamine (2.47)	0.09644 ± 0.01241	0.09548 ± 0.01768	0.72204
Pyruvate (2.365)	0.03074 ± 0.00577	0.02922 ± 0.00602	0.14573
Lipid (2.23)	0.12925 ± 0.08064	0.12036 ± 0.06102	0.47984
N-acetylglycoprotein (2.054)	0.32462 ± 0.11493	0.31241 ± 0.08730	0.49632
Lipid (2.01)	0.34895 ± 0.11742	0.34877 ± 0.13591	0.99377
Acetate (1.915)	0.02723 ± 0.00443	0.02956 ± 0.01926	0.34458
Lipid (1.6)	0.25874 ± 0.16146	0.24437 ± 0.12969	0.57693
Lipid	0.24517 ± 0.14945	0.21435 ± 0.12022	0.19762
Lactate (1.332)	0.51526 ± 0.21607	0.54140 ± 0.20130	0.47661
Lipid (1.31)	3.17346 ± 1.64186	2.92170 ± 1.37389	0.34491
Valine (1.044)	0.03794 ± 0.01059	0.03926 ± 0.01097	0.48680
Isolecuine (1.005)	0.01209 ± 0.00389	0.01224 ± 0.00349	0.82135
Leucine (0.965)	0.02646 ± 0.00641	0.02479 ± 0.00567	0.11783
Lipid (0.90)	1.59928 ± 0.50784	1.42274 ± 0.44536	0.03709

Data are presented as mean ± SD. *VIP* variable importance in the projection

## Discussion

It is reported hormonal and sex chromosome mechanisms may interact in the sex-specific control of certain diseases such as atherosclerosis, aneurysms, ischemia/reperfusion injury, and hypertension [23], and gender and age were identified as the principal factors explaining metabolome variability [24]. Since our CAS patients tended to be older and were mostly males, but the controls were relatively younger, we recruited only male subjects for study and did our best to keep the age balanced between controls and CAS patients (*p* value = 0.050). It has been reported that diabetes may have far reaching metabolic effects beyond raised blood glucose and may impact systemic metabolism [25]. To prevent from bias due to gender and diabetes in metabolomics study, our study recruited only males and excluded patients with diabetes.

Our study has its novelty since the etiologies of atherosclerosis is multifactorial, we combined metabolomics analysis with the traditional risk factors to identify the biomarkers for CAS. We found CAS patients had lower choline but higher homocysteine and lipid levels compared to controls. Decreased serum levels of several individual phosphatidylcholine and lysophosphatidylcholine species were observed in the patients with peripheral artery disease and CAD in comparison to the healthy subjects [26]. Polymorphic variation in the choline transporter (CHT1) gene was reported to predict early, subclinical measures of carotid atherosclerosis [27]. The low dietary intake of choline and its metabolite betaine may aggravate the atherogenesis through the effects on homocysteine methylation pathways as well as the choline’s antioxidants properties [28]. Elevated plasma homocysteine and lipid levels are well known risk factors for atherosclerosis [29]. In the investigation of early-stage atherosclerotic development and progression in chow-fed apolipoprotein E-deficient mice, distinct plasma metabolomic profiles of glycerophospholipid and sphingolipid may help to differentiate the different stages of atherosclerotic progression [30]. The study of carotid plaque tissue using two ultra performance liquid chromatography coupled to mass spectrometry (MS) found changes in several metabolite species were consistent with well-established pathways in atherosclerosis such as the cholesterol, purine, pyrimidine, and ceramide pathways [31]. Also, metabolites related to the eicosanoid pathway (arachidonic acid and arachidonic acid precursors) and the three acylcarnitine species (butyrylcarnitine, hexanoylcarnitine, and palmitoylcarnitine) that act as intermediates of the beta-oxidation were detected in higher intensities in symptomatic carotid plaque tissues compared to asymptomatic tissues [32]. These data in combination with ours may suggest these metabolites especially plasma choline, homocysteine, and lipid profiling may be used as biomarkers for atherogenesis.

It is suggested that NMR metabolomics can be incorporated as a routine survey in large biobanks, which is beneficial both from the inexpensiveness and reproducibility [33, 34]. Successful examples include NMR metabolic profiling of cardiovascular events [35–38] and ischemic stroke [39]. Stegemann et al. found three lipid species (triacylglycerols, cholesterol esters, and phosphatidylethanolamines) on top of traditional risk factors could improve risk discrimination and classification for cardiovascular disease [37]. Wurtz et al. reported tyrosine and glutamine levels were cross-sectionally associated with carotid intima media thickness and the presence of angiographically ascertained CAD in independent populations [36]. Review article by Qureshi et al. demonstrated certain metabolic pathways may be related to the pathological processes of stroke, particularly including branched chain amino acid, homocysteine, folate, anaerobic, and lipid metabolism [39]. Levels of plasma branched-chain amino acid, measured by NMR, were found elevated in type 2 diabetes and could be associated with carotid intima media thickness, a proxy of subclinical atherosclerosis [40]. Brindle et al. found NMR-based profiling had significant difference and provided a > 90% predictive power for discrimination between subjects with severe CAD and those with angiographically normal coronary arteries [4].

In the study of cardiovascular disease using MS, the use of liquid chromatography-MS found lactate, byproducts of AMP metabolism, and metabolites of the citric acid cycle were discordant between the individuals with and without exercise-induced myocardial ischemia [9]. The MS/MS-based studies found two PCA-derived metabolite factors were able to discriminate individuals with CAD from those without CAD [6], and there was a strong association of arginine and its downstream metabolites, ornithine and citrulline, with CAD and with major adverse cardiovascular events including death, myocardial infarction, and stroke [5]. In patients undergoing cardiac catheterization, five metabolites were found to be independently associated with mortality including medium-chain acylcarnitines, short-chain dicarboxylacylcarnitines, long-chain dicarboxylacylcarnitines, branched-chain amino acids, and fatty acids [41]. It is postulated that metabolic profiles can help to predict cardiovascular events independently of standard predictors.

In the study of stroke, patients with stroke recurrence were found to have significantly lower concentrations of a specific lysophosphatidylcholine (LysoPC [16:0]) [42]. Moreover, LysoPC (20:4) was also identified to be a potential biomarker of stroke recurrence and might increase the prediction power of age, blood pressure, clinical features, duration of symptoms, diabetes scale, and large artery atherosclerosis. In the cases of large artery atherosclerosis, a potential biomarker of LysoPC (22:6) was also suggested [42]. In the studies of ischemic stroke process, Wang et al. [43] found 13 metabolites had significant changes with malonic acid and glycine being the most noticeable variable metabolites. This dramatic change of malonic acid and glycine was suggested to be able to serve as biomarkers in the dynamic pathogenesis of cerebral ischemia. In the study of acute ischemic stroke, increased plasma excretion of lactate, pyruvate, glycolate, and formate, decreased excretion of glutamine and methanol, and decreased urine levels of citrate, hippurate, and glycine were noticed in stroke patients compared to healthy controls [13]. These metabolites detected from plasma and urine of patients with acute cerebral infarctions were suggested to be associated with anaerobic glycolysis, folic acid deficiency, and hyperhomocysteinemia.

## Conclusions

Many previous studies have demonstrated the significance of metabolomics in the prediction of cardiovascular and cerebrovascular diseases. However, the study of CAS is limited. In CAS, there remains a clinical need to develop an objective test to confer rapid and accurate diagnostic discrimination of atherosclerosis to enable the provision of better direct investigation for rapid intervention. The present study showed choline, homocysteine, and lipids in association with traditional risk factors could be predictive biomarkers for carotid artery atherosclerosis. Previous genome-wide association studies have demonstrated certain genes are associated with extracranial carotid artery atherosclerosis. The use of metabolomics in the biomarker research of extracranial carotid atherosclerosis may add the potential to improve the accuracy of diagnostic discrimination of genomic predictors in atherogenesis.

## Additional file

Additional file 1: **Figure S1.** The S-plot for orthogonal-partial-least-squares-discriminant-analysis (OPLS-DA) run by MetaboAnalyst 4.0 in control group and carotid artery stenosis group. (TIF 230 kb)

## Data Availability

The datasets used and/or analysed during the current study are available from the corresponding author on reasonable request.

## Abbreviations

CAD: Coronary artery disease
CAS: Carotid artery stenosis
MS: Mass spectrometry
