# Supplementary figures and images for: Metabolomics study in severe extracranial carotid artery stenosis
Source: BMC Neurol. 2019 Jun 24;19:138. doi: 10.1186/s12883-019-1371-x (PMC6589885; doi:10.1186/s12883-019-1371-x)

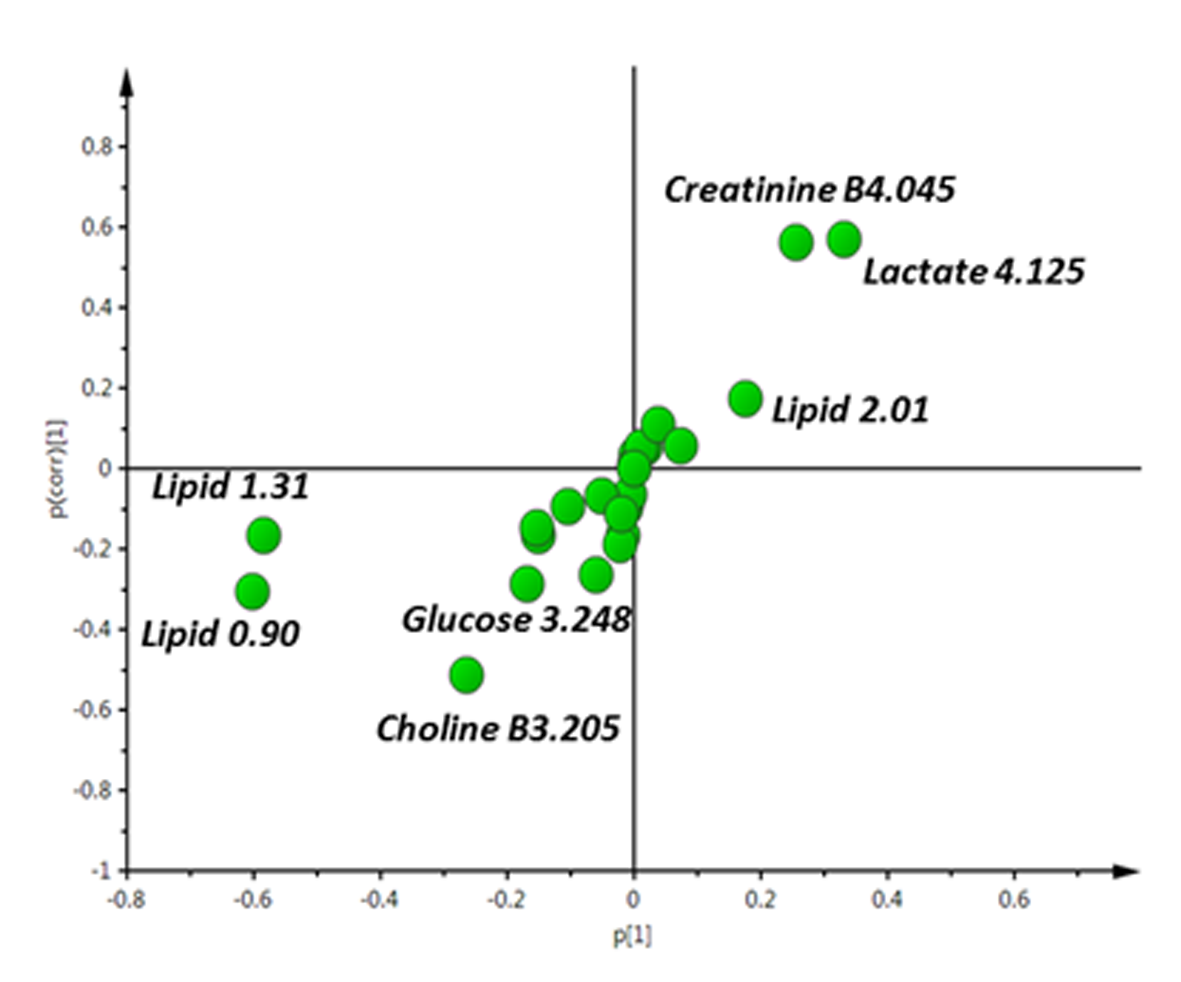

Supplement: Supplementary file 1 — Figure S1. The S-plot for orthogonal-partial-least-squares-discriminant-analysis (OPLS-DA) run by MetaboAnalyst 4.0 in control group and carotid artery stenosis group. (TIF 230 kb) [file 12883_2019_1371_MOESM1_ESM.tif]
